# Supplementary material for: The Development and Testing of a Patient Decision Aid for Individuals with Homologous Recombinant Proficient Ovarian Cancer Who Are Considering Niraparib Maintenance Therapy
Source: Curr Oncol. 2024 Mar 8;31(3):1416–25. doi: 10.3390/curroncol31030107 (PMC10969309; doi:10.3390/curroncol31030107)
Supplement: Supplementary file 1 [file curroncol-31-00107-s001.zip › curroncol-2874883-supplementary.pdf]

Patient Decision Aid on Niraparib Treatment – Patient Survey

Please circle the best answer to each question.

1) The language used was easy to follow.

Strongly Disagree	Disagree	Neutral	Agree	Strongly Agree

Comment: ____

2) The amount of information provided was:

Much less than wanted	Little less than wanted	About right	Little more than wanted	Much more than wanted

Comment: ____

3) The length of the decision aid was:

Too short	Just right	Too long

Comment:____

4) The Benefits and Harms sections were easy to follow:

Strongly Disagree	Disagree	Neutral	Agree	Strongly Agree

Comment:____

5) I found the decision aid was:
   - ☐ Slanted towards treatment
   - ☐ Slanted towards no treatment
   - ☐ Well balanced

6) I would recommend this decision aid for patients who want more information about taking Niraparib.

Strongly Disagree	Disagree	Neutral	Agree	Strongly Agree

Comment:____

7) I am satisfied with the overall quality of this patient decision aid.

Strongly Disagree	Disagree	Neutral	Agree	Strongly Agree

Comment:____

8) What did you like about the patient decision aid?

____

____

9) Do you have any other suggestions for information that would be helpful to include on the patient decision aid?

____

____

Version date: October 19th, 2022

Patient Decision Aid on Niraparib Treatment – Physician Survey

Please circle the best answer to each question.

1) The language used was easy to follow.

Strongly Disagree	Disagree	Neutral	Agree	Strongly Agree

Comment: ____

2) The amount of information provided was:

Much less than wanted	Little less than wanted	About right	Little more than wanted	Much more than wanted

Comment: ____

3) The length of the decision aid was:

Too short	Just right	Too long

Comment:____

4) The Benefits and Harms sections were easy to follow:

Strongly Disagree	Disagree	Neutral	Agree	Strongly Agree

Comment:____

5) I found the decision aid was:
   - ☐ Slanted towards treatment
   - ☐ Slanted towards no treatment
   - ☐ Well balanced

6) I would recommend this decision aid for patients who want more information about taking Niraparib.

Strongly Disagree	Disagree	Neutral	Agree	Strongly Agree

Comment:____

7) I am satisfied with the overall quality of this patient decision aid.

Strongly Disagree	Disagree	Neutral	Agree	Strongly Agree

Comment:____

8) What did you like about the patient decision aid?

____

____

9) Do you have any other suggestions for information that would be helpful to include on the patient decision aid?

____

____

Version date: October 19th, 2022
